# Dietary Diversity and Its Associated Factors Among High School Adolescent Girls in Addis Ababa, Ethiopia, 2025

**DOI:** 10.1002/puh2.70340

**Published:** 2026-08-13

**Authors:** Bekalu Getachew Gebreegziabher, Aragaw Gizaw Shumete, Habtamu Molla Ayele, Ruth Tilahun Nida, Abdu Oumer

**Affiliations:** ^1^ School of Public Health Menelik II Medical and Health Science College Addis Ababa Ethiopia; ^2^ Department of Planning, Monitoring and Evaluation Kombolcha General Hospital Kombolcha Amhara Ethiopia; ^3^ Maternal and Child Health Directorate Federal Ministry of Health Addis Ababa Ethiopia; ^4^ College of Health Sciences, Department of Midwifery Dilla University Dilla Ethiopia

**Keywords:** Addis Ababa, adolescent girls, dietary diversity, Ethiopia

## Abstract

**Background:**

Dietary diversity (DD) assesses the access and consumption of varieties of food types, there are few studies conducted on DD, the current study targeted adolescent girls in high schools in Addis Ababa, Ethiopia.

**Objective:**

To assess the magnitude of DD and its associated factors among high school adolescent girls in Addis Ababa, Ethiopia.

**Methods:**

An institutional‐based cross‐sectional study was conducted on 621 female adolescents from May 1 to 30, 2025. A multistage sampling technique was used to select study participants. The data were entered into epi‐data version 3.1 and were exported to SPSS version 25. Variables were screened using the bivariable logistic regression analysis model. Those candidate variables having *p* value ≤ 0.25 were exported to the multivariable logistic analysis. Statistical significance was declared if *p *< 0.05.

**Result:**

The prevalence of inadequate dietary diversity score (DDS) among adolescent girls was 33.4%, 95%CI [29.66%, 37.14%] with average of 6.48 food groups consumed daily. Grains/roots and tubers (77.7%) were the most commonly consumed food groups in the last 24 h dietary recall. Maternal education [adjusted odds ratio (AOR) = 1.42, 95%CI, (1.12–2.99)], Meal frequency [AOR = 1.54, 95%CI, (1.06–2.25)], Nutritional education [AOR = 1.53, 95%CI, (1.35–1.80)] and Consumption of sweet foods [AOR = 1.71, 95%CI, (1.17–2.50)] were independent predictors of inadequate DDS.

**Conclusion:**

One third of female adolescents had inadequate DDS. Grains/tubers and roots were the most consumed food groups. Maternal education, meal frequency, nutritional education, and nonconsumption of more sweet foods/drinks were significantly associated with the inadequate DDS.

## Graphical abstract

Dietary diversity among adolescent girls remains suboptimal, with many consuming limited food groups. Key factors influencing diversity include meal frequency, sweet food consumption, maternal education, and nutrition education. Low diversity increases the risk of micronutrient deficiencies, highlighting the need for targeted school‐based nutrition interventions and improved access to diverse, nutrient‐rich foods.

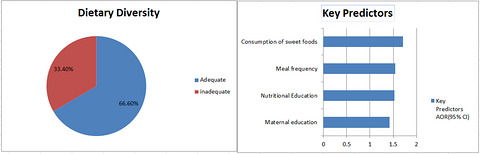

## 1 Introduction

Adolescence is a period of rapid growth and development in a human's life; during this, proper nutrition is vital for healthy growth, development, sexual maturation, and future health [1]. Dietary diversity (DD) describes the number of different food groups consumed over a given time period [2]. It qualitatively measures the access and the consumption of varieties of food groups as a proxy for nutrient adequacy and quality at individual and household levels [3]. It focuses on diversifying one's diet by including different food items. It is vital for adolescents to achieve and maintain nutrient adequacy for their optimal growth and development [4]. The dietary diversity score (DDS) is a cost effective tool for quantifying DD, and it was initially developed by the Food and Agriculture Organization (FAO) of the United Nations and used with a single 24‐h dietary recall by counting the number of food groups rather than foods consumed [5] according to a recent research evidence, only 17% of adolescents had diversified diet [6].

Adolescent girls only had few windows of opportunities to break the vicious cycle of malnutrition [7]. The problem of malnutrition and its causes in developing country like Ethiopia is deep rooted [8]. According to a study done in northeast part of Ethiopia, the prevalence of inadequate DD among adolescents in Woldia Secondary Schools was 49.1% [9]. Many studies have shown that an inadequately diversified diet characterized by the deficiency of macronutrients and micronutrients results in poor nutritional status, especially in schoolchildren and adolescent girls from developing countries [10, 11].

Girls are more vulnerable to nutritional problems compared to boys because of the biological differences [12]. Dietary habits of adolescent girls vary considerably due to their influence by their peers, exposure and influence of mass media, and their body image. Adolescents tend to snack and graze together, skip their meals for several reasons, practice out of home eating, eat at late hours, and usually consume readymade fast foods more frequently [13]. Usually monotonous staple diets among adolescent girls lack the essential micronutrients, leading to macro and micronutrient deficiencies [14]. A suboptimal dietary intake among adolescent girls was 45%–60% [15], as a consequence, several micronutrient deficiencies may result, including vitamin A and iodine deficiencies [16, 17]. A research evidence shows that delayed puberty, contracted pelvis, and unfavorable birth outcomes were the consequences in undernourished adolescent girls [18].

Nowadays adolescent girls social media use is increasing as a recent study showed that majority of girls (58%) are more interested to lose their weight for several reasons and only 9% of adolescent girls loves to gain weight and a great proportion 85% of young women worry about their body shape and looks [19].

The adolescent girls were not targets of nutritional interventions previously; in contrast, this is the most important stage for physical and mental development. Different studies have shown that psychosocial, sociodemographic status, and diet‐related behavior are predictors of DDSs [20]. Behavioral factors like inadequate physical activity, smoking, and alcohol drinking practiced at the time of adolescence can influence and be influenced by nutrition [21]. A national report shows that 29% and 30% of adolescent girls were found to have thinness and anemia, respectively [22, 23]. Local evidence shows that many adolescent girls enter pregnancy with poor nutritional status. They may have iron deficiency anemia and other adverse feto‐maternal outcomes like low birth weight and neural tube defects [16].

In Ethiopia, research was done on predictors of DD among under‐five children and pregnant and lactating women, but there is limited evidence among adolescent girls’ dietary practices. Even though the city government of Addis Ababa has launched extensive school feeding programs (SFPs) in all government primary schools aimed at averting school hunger and improving the nutrition status of schoolchildren, but this SFP did not include the secondary schools. Moreover, the adolescent period is a critical age in which nutritional requirements are high due to fast growth and development. So, the study aimed to assess the level of DD and associated factors among adolescent girls attending high schools in Addis Ababa, Ethiopia.

## 2 Methods and Materials

### 2.1 Study Design and Setting

#### 2.1.1 Study Setting

The study was conducted in Addis Ababa, Ethiopia. There are 11 sub‐cities and 129 woredas in Addis Ababa, the study was conducted in secondary schools of Addis Ababa. According to report of Addis Ababa City Administration Education Bureau (AACAEB), there are 218 general secondary schools [9, 10, 11, 12]. From those schools, 72 are government, and 146 are nongovernment schools. The study was conducted in four sub‐cities of Addis Ababa, namely, Bole sub‐city, Yeka sub‐city, Lemi Kura sub‐city, and Akaki Kality sub‐city. Lemi Kura has more private high schools than any other sub‐city [16], followed by Bole [18], Yeka [9], and Akaki Kality [8]. On the other hand, Akaki Kality has the greatest number of government high schools [11], followed by Lemi Kura [10], Bole [8], and Yeka [7]. In all, among the four sub‐cities, there are far more private secondary schools [53] than government secondary schools [33] (Table 1).

**TABLE 1:** Number of high schools and female students in selected sub‐cities of Addis Ababa, Ethiopia, 2024.

Sub‐cities	Number of high schools		Number of female students	
	Private	Government	Private	Government
Akaki Kality	8	11	3420	7809
Bole	18	8	3503	5371
Lemi Kura	24	10	5057	8663
Yeka	9	7	2978	8902
Total	59	36	14,958	30,745

### 2.2 Study Period

The study was conducted from May 1 to 30, 2025.

### 2.3 Study Design

A school‐based cross‐sectional study was conducted.

### 2.4 Population

#### 2.4.1 Source Population

All adolescent girls (aged 10–19) enrolled in a governmental and private secondary school (9–12 grade) in the academic year of 2017 in Addis Ababa.

#### 2.4.2 Study Population

All adolescent girls from selected sub‐cities in private and governmental secondary schools in the academic year of 2017 E.C. in Addis Ababa were the study population.

### 2.5 Eligibility Criteria

#### 2.5.1 Inclusion Criteria

All adolescent girls attending secondary schools in Addis Ababa during the study period.

#### 2.5.2 Exclusion Criteria

- All adolescent girls whose ages are greater than or equal to 20 years.
- Pregnant adolescents.
- Girls with established chronic illness.
- Girls who declined to participate or for whom informed consent or assent was not obtained were excluded from the study.

### 2.6 Sample Size Determination

The sample size was calculated for each objective separately. Therefore, the sample size was estimated using the single population proportion formula, 95%CI, 5% margin of error by taking the proportion of 43.3% from DDS among adolescent girls in Yeka sub‐city [24]:

$$n={\left(Z\alpha /2\right)}^{2}\times p\times \left(1-p\right)/d^{2}$$

$$n={\left(1.96\right)}^{2}\times 0.433\times 0.567/{\left(0.05\right)}^{2}$$

n=0.943/0.0025

n=377

Considering the design effect of 1.5% and 10% nonrespondent rate the final sample size was 621.

### 2.7 Sampling Procedure

**FIGURE 1:**
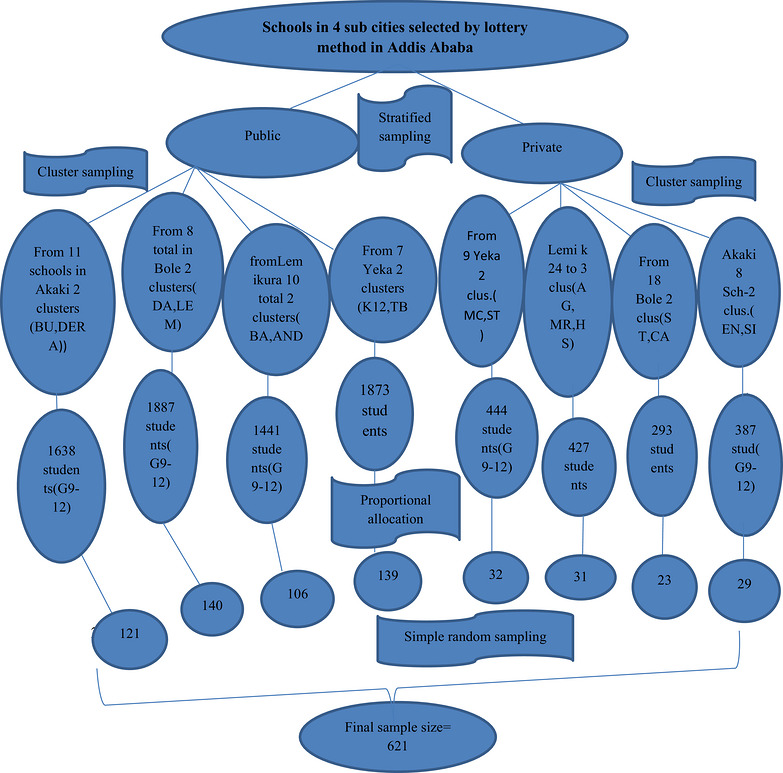
Sampling technique of high school adolescent girls in Addis Ababa, Ethiopia, 2025.

Multistage cluster sampling technique was used to select the study participants. The existing high schools were stratified to public and private schools. A lottery method was used to select schools from the government and private schools. The number of female students from the selected clusters/schools was determined by using proportionate sampling technique. Finally, adolescent girls were selected from government and private schools by using simple random sampling technique (Figure 1).

#### 2.7.1 Study Variables

##### 2.7.1.1 Dependent Variable

- Level of DD (adequate or inadequate).

##### 2.7.1.2 Independent Variables

- Sociodemographic factors: Age, grade level, family size, mother's education, and occupation.
- Nutritional and meal pattern‐related factors: Nutrition education, source of food, home gardening, nutritional knowledge, source of information, meal frequency, eating companion, meal skipping, and eating out of home.
- Health and sanitation related: History of chronic disease, history of diarrhea, alcohol use, khat chewing, cigarette smoking, and physical activity.
- Psychosocial factors: Long stay on social media, fear of obesity, avoidance of food, stress/anxiety/worried, family disagreement, reduced food due to cultural impact, and missed food due to education.

#### 2.7.2 Instrument

Data were collected using a semi‐structured, self‐administered questionnaire developed after reviewing relevant literature and previously validated tools [2, 9, 14, 25, 26, 27]. The questionnaire consisted of five sections: (1) sociodemographic characteristics, (2) nutritional and meal patterns, (3) health‐ and sanitation‐related factors, (4) psychosocial factors, and (5) DD assessment.

The sociodemographic section included 12 items adapted from prior adolescent nutrition studies conducted in Ethiopia. Nutritional and meal pattern variables (16 items), health and sanitation factors (8 items), and psychosocial variables (7 items) were adapted from similar studies conducted in Jimma and Nifas Silk Lafto sub‐city [40, 48], with minor contextual modifications to reflect the study setting.

DD was assessed using the FAO Minimum Dietary Diversity for Women (MDD‐W) guideline [5]. The tool includes 10 standardized food groups and uses a 24‐h recall method, where participants reported all foods and beverages consumed in the previous day. The DDS was calculated by summing the number of food groups consumed (range: 0–10). Participants consuming five or more food groups were categorized as having adequate DD, whereas those consuming fewer than five were categorized as having inadequate DD [3]. Although originally designed for women of reproductive age, the MDD‐W tool has been widely applied in adolescent populations in similar contexts. The tool was reviewed and slightly adapted to reflect locally available foods without altering the core food group classification.

Nutritional knowledge was assessed by questions that intend to explore the cause and prevention mechanism of selected nutritional problems. Dietary awareness question was adapted from a similar study conducted in Jimma [28] and Nifas Silk Lafto [29]. It consists of 16 closed‐ended question items. The overall nutritional knowledge score was determined by adding the responses; each correct answer received “1” point, whereas the incorrect response received “0” points. For knowledge, the maximum and minimum scores were 16 and 0, respectively. That is, values above the mean will be recorded as “1,” which indicates good nutritional knowledge, and values below the mean will be coded as “0,” which indicates poor nutritional knowledge.

### 2.8 Data Collection Procedure

A semi‐structured, self‐administered questionnaire was used. Prior to data collection, schools were stratified to private and government. Representative schools were selected from total number of schools (clusters). The questionnaire was prepared in Amharic language. Before data collection, a pretest was done on 31 participants. DD of adolescent girls was based on FAO 10 food groups as all foods and beverages consumed by the respondents in the past 24 h were asked. Participants completed the questionnaire in the classroom under supervision of trained data collectors.

### 2.9 Operational and Term Definition

**DDS**: measures the number of food groups consumed over a 24 h period and adolescent who consumed less than 5 food groups out of 10 [10] were categorized to have inadequate DD, whereas those who consumed five and above food group will be considered to have an adequate DD [21].

**Adolescents**: Refers to female adolescents aged 10–19 years [30].

**Inadequate DD**: If the respondents have consumed less than 5 foods from the 10 food groups.

**Adolescent nutritional knowledge**: Respondents whose knowledge scores equal the mean and are above were categorized as having good knowledge, whereas respondents whose knowledge scores are below the mean score were categorized as having poor nutritional knowledge [18].

**Long stay on social media**: If an adolescent girls stays more than 2 h on social media (Facebook, Twitter, Telegram, and TikTok) in a day [24].

**Sweet foods/beverages**: Include all food items with a high content of different sweetening agents (e.g., sugar, corn syrup, other syrup, honey, molasses, or jaggery), such as biscuits (sweet), cakes, candies (hard candies, toffees, “milk toffees,” or candies made with sweetened condensed milk, any other candies), chocolates, coconut candies and sweet biscuits, and other sweetened coconut snacks, cookies, frozen custard/frozen yoghurt, fruit canned in sugar syrup fruit “gummy” candies, fruit “leathers,” Ice cream, halwa, honey, and sweet beverages includes chocolate drinks, fortified and unfortified, both prepackaged fluid drinks and powders, coffee with sugar, energy drinks, fruit drinks, sweetened fruit juices, malt drinks, fortified and unfortified, soft drinks/sodas/carbonated or “fizzy drinks,” including colas, fruit flavors and other flavors, tea with sugar, any other drink sweetened with sugar, corn syrup, honey, or other sweetener [5].

**Alcohol use**: Ever used if adolescent girls drink any form of alcoholic products (Tej, Tella, Beer, and Areke) at least once in their lifetime. Current use if they have drunk alcohol in the last 7 days [31].

**Cigarette smoking**: Ever smoked if the girl ever smoked or even tried taking one or more puffs at least once in their lifetime. Current use if the girl smoked ≥1 cigarette in any of the last 7 days [32].

**Khat use**: If adolescent girls chewed khat at least once in their lifetime. Current use if they chewed once in the last 7 days [33].

**Consumption of more sweet foods and drinks**: If the girls consume sweet foods/beverages more than four times per week [34].

**Source of income**: Families of adolescent girls who reported receiving income on a regular schedule (e.g., weekly/monthly allowance or stipend) were classified as having a fixed income. Those who received income irregularly, such as through daily labor, gifts, or sporadic business activities, were categorized as having a non‐fixed income [35].

**Some food avoidance**: Intentional avoidance of consuming one or more specific food items or food groups for any reason (gender, religious, health, or personal preference) during the past 30 days [36].

**Family history of chronic disease**: An adolescent girl was considered to have a family history of chronic disease if she reports that at least one of her first‐degree relatives (parents or siblings) or second‐degree relatives (grandparents, aunts, or uncles) has ever been diagnosed with a chronic non‐communicable disease (such as diabetes, hypertension, renal disease, cancer, asthma, or heart disease) by a health professional [37].

**Physical activity**: Adolescent girls were considered to be physically active if they perform walking, running, playing sports, dancing, active play performed for at least 60 min per day. Physically inactive if she did not meet the above criteria [38].

### 2.10 Data Quality Assurance

Data were collected using pretested and properly designed questionnaires. The internal consistency of the tools was checked by using the Cronbach's alpha test, and it was found to be at the acceptable level (0.78). To assure data quality, the questionnaires were first prepared in English, then translated first into Amharic and then retranslated back to English to check the consistency. Pretesting of the questionnaires was performed using 5% [28] of the sample size on high school students in Shaggar City with similar sociodemographic characteristics. On the basis of the pretest, some modifications were done to the questionnaire. Orientation was given to supervisor on the data collection tools, data collection techniques, and maintaining of confidentiality of the respondent for 1 day. During the actual data collection, every day after data collection, questioners were reviewed and checked for completeness, word errors, unclear questions, and consistency by the principal investigator, and the necessary feedback was given to data collectors each morning.

### 2.11 Data Analysis

All data were checked, coded, and entered into Epi‐data version 3.1 and exported into SPSS version 25 software packages for analysis. Descriptive statistics were calculated. The association between independent and dependent variables was assessed using odds ratio with 95% confidence interval. Pseudo regression was done to check multicollinearity between independent variables, and the result of variance inflation factor was less than five (no multicollinearity was detected). A bi‐variable logistic regression analysis was run to select candidate variables for multivariable analysis. Variables with *p* value <0.25 were taken as a cutoff point to select eligible variables for the multivariable logistic regression analysis. Multivariable logistic regression models were fitted, and the adequacy of the model to predict the outcome variables was checked by Hosmer–Lemeshow goodness‐of‐fit and *p* value <0.05 was declared as a statistically significant in the final model. Tables and text were used to present the result.

### 2.12 Ethical Considerations

Ethical approval was obtained from Menelik II Medical and Health Science College and Addis Ababa Health Bureau research ethics committee with ethical clearance number (AAHB/072/17) prior to data collection. Support permission was also obtained from respective school administrations.

A written informed consent was obtained from the parents or legal guardians of adolescent girls aged under 18 years, whereas written assent was obtained from the adolescent girls themselves. For participants aged 18–19 years, written informed consent was obtained.

Information regarding the study objectives, procedures, potential risks, and benefits was clearly explained to both participants and their guardians of the adolescent girls through an information sheet provided in the local language (Amharic). The study participants were informed that their participation was completely voluntary, and they had the right to withdraw from the study at any time without any consequences. Confidentiality and anonymity of the collected data were strictly maintained throughout the study.

## 3 Results

### 3.1 Sociodemographic Characteristics of Respondents

A total of 610 female adolescents were participated in the study, providing a response rate of 98.2%. The mean ± SD age of the participants was 17.02 ± 1.208 years. Regarding age distribution, the majority, 411 (67.4%), of girls were in the age range of 17–19 years. Almost two–thirds, 399 (65.4%), of the participants were enrolled in government Schools. Half, 306 (50.2%), of the female students were from grade 11 and 12. About 271 (44.4%) of respondent mothers/caretakers were housewives. Family size was less than five in 386 (63.3%) of the participants. More than two–fifths, 255 (41.8%), of mothers/caretakers have attended secondary education and above. Fixed income was reported to be 368 (60.3%). Fathers were found to be the head of the households in 312 (51.1%) of the households. The majority, 195 (32%), of fathers were government employees (Table 2).

**TABLE 2:** Sociodemographic characteristics of adolescent girls attending secondary schools in Addis Ababa, Ethiopia, 2025.

Variable (*n* = 610)		Frequency (%)
Age (in years)	14–16	199 (32.6)
	17–19	411 (67.4)
School type	Government	399 (65.4)
	Private	211 (34.6)
Grade	9–10	304 (49.8)
	11–12	306 (50.2)
Mothers’ occupation	Governmental	67 (11)
	Nongovernmental	121 (19.8)
	Merchant	121 (19.8)
	Housewife	271 (44.4)
	Others^a^	30 (4.9)
Family size	<5	386 (63.3)
	≥5	224 (36.7)
Mothers’ education	Cannot read and write	61 (10)
	Read and write	130 (21.3)
	Primary education	164 (26.9)
	Secondary and above	255 (41.8)
Fathers’ education	Cannot read and write	35 (5.7)
	Read and write	117 (19.2)
	Primary education	144 (23.6)
	Secondary and above	314 (51.5)
Family income	<11,453 ETB	408 (66.9)
	≥11,453 ETB	202 (33.1)
Source of income	Fixed	368 (60.3)
	Non‐fixed	242 (39.7)
Fathers’ occupation	Governmental	195 (32)
	Nongovernmental	123 (20.2)
	Merchant	86 (14.1)
	Private	160 (26.2)
	Retired	26 (4.3)
	Others^b^	20 (3.3)
Head of household	Mother	168 (27.5)
	Father	312 (51.1)
	Mother and father	22 (3.6)
	Brother	43 (7)
	Caretaker	31 (3.1)
	Sister	34 (5.6)

Abbreviation: ETB, Ethiopian Birr.

^a^Beauty shop, Chef.

^b^Farmers.

### 3.2 Nutritional and Meal Pattern–Related Characteristics

The majority, 369 (60.5%), of adolescent girls have had eating companions, and about 231 (37.7%) ate with their family members. More than four–fifths, 513 (84.1%), of female adolescents’ food source was from home cooking. Only 237 (38.9%) of participants missed their meal in the last 24 h. Lunch was the most commonly missed meal in 111 (18.2%) of the participants. More than two fifth 251 (41.1%) of adolescent girls had a home gardening. Less than one‐third, 194 (31.3%), of girls had out of home eating practice. About 387 (63.4%) of girls ate breakfast before school. Only 191 (31.3%) of adolescent girls had taken nutritional education. The majority, 507 (83.1%), of girls had taken 3–4 meals per day. The mean meal frequency was 3.48 ± 0.84 (mean ± SD). More than half, 326 (53.4%), of female adolescents prefer to consume more sweets. The mainstream media, 197 (32.3%), is the main source of information about DD. Mothers, 294 (98.2%), were found to be the main decision makers for meal preparation (Table 3).

**TABLE 3:** Nutritional and meal pattern–related characteristics of adolescent girls attending secondary schools in Addis Ababa, Ethiopia, 2025.

Variables (*n* = 610)		Frequency (%)
Eating companion	Yes	369 (60.5)
	No	241 (39.5)
Eating with	Family	231 (37.9)
	Friends	125 (20.5)
	Others	13 (2.1)
Source of food	Own	513 (84.1)
	Food aid	77 (12.6)
	Purchase	20 (3.3)
Missed meal in last 24 h	Yes	237 (38.9)
	No	373 (61.1)
Type of missed meal	Breakfast	51 (8.4)
	Lunch	111 (18.2)
	Dinner	75 (12.2)
Home gardening	Yes	251 (41.1)
	No	359 (58.9)
Eating out of home	Yes	194 (31.8)
	No	416 (68.2)
Eating out frequency/week	Once	46 (7.5)
	Twice	64 (10.5)
	Three	39 (6.4)
	Four and above	43 (7.4)
Eats breakfast before school	Yes	387 (63.4)
	No	223 (36.6)
Reason for missed breakfast	Breakfast not prepared	41 (6.7)
	Prefer eating out of home	79 (13)
	Fear of being late for school	39 (6.4)
	Don't like to eat early morning	64 (10.5)
Nutritional education	Yes	191 (31.3)
	No	419 (68.7)
Meal frequency per day	1–2	61 (10.1)
	3–4	507 (83.1)
	>4	42 (6.9)
Consumption of sweet foods	Yes	326 (53.4)
	No	284 (46.6)
Information about DD	No information	139 (22.8)
	Mainstream media	197 (32.3)
	School	191 (31.3)
	Friends	45 (7.4)
	Family	25 (4.1)
	Social media	13 (2.1)
Snacking	Yes	427 (70)
	No	183 (30)
Decision maker on meal	Mothers	294 (98.2)
	Father	85 (13.9)
	Mother and father	95 (15.6)
	Sister	33 (5.4)
	Brother	23 (3.8)
	Me	45 (7.4)
	Uncle and aunts	35 (5.7)

Abbreviation: DD, dietary diversity.

### 3.3 Psychosocial Characteristics

More than two–thirds, 409 (67%), of female adolescents worry about their shape/gaining weight. Only 195 (32%) of adolescents reported a family disagreement. More than half, 340 (55.7%), of adolescent girls had stayed longer on social media. More than two–fifths, 280 (45.9%), of adolescent girls had avoidance of some foods (Table 4).

**TABLE 4:** Psychosocial characteristics of adolescent girls attending secondary schools in Addis Ababa, Ethiopia, 2025.

Variables (*n* = 610)		Frequency (%)
Fear of obesity/worry about shape	Yes	409 (67)
	No	201 (33)
Family disagreement	Yes	195 (32)
	No	415 (68)
Anxiety/Depression	Yes	286 (46.9)
	No	324 (53.1)
Long stay on social media	Yes	340 (55.7)
	No	270 (44.3)
Loss of appetite	Yes	235 (38.5)
	No	375 (61.5)
Some foods avoidance	Yes	280 (45.9)
	No	330 (54.1)
Cultural food avoidance	Yes	127 (20.8)
	No	483 (79.2)

### 3.4 Health‐ and Sanitation‐Related Characteristics

More than one–tenth, 97 (15.9%), of adolescent girls had a history of diarrhea in the last 24 h only 134 (22%) of girls had a family history of chronic disease. Only 18 (3%) and 5 (0.8%) of adolescent girls had ever used alcohol and khat, respectively. Half of 306 (50.2%) of the adolescents have done physical exercise (Table 5).

**TABLE 5:** Health‐ and sanitation‐related characteristics of adolescent girls attending secondary schools in Addis Ababa, Ethiopia, 2025.

Variables (*n* = 610)		Frequency (%)
History of diarrhea	Yes	97 (15.9)
	No	513 (84.1)
Family history of chronic disease (DM, HTN)	Yes	134 (22)
	No	476 (78)
Hand washing practice	Yes	603 (98.9)
	No	7 (1.1)
Ever/Current used alcohol	Yes	18 (3)
	No	592 (97)
Ever used khat	Yes	5 (0.8)
	No	605 (92.8)
Ever smoked	Yes	2 (0.3)
	No	608 (99.7)
Physical activity	Yes	306 (50.2)
	No	304 (49.8)
Number of minutes/day	Active	69 (22.5)
	Inactive	237 (77.4)

### 3.5 Nutritional Knowledge

The mean knowledge score of adolescent girls was found to be 10.25 ± 3.04 (mean ± SD). About 307 (50.3%) of female adolescents had poor nutritional knowledge. About 328 (53.8%) of female adolescents knew the dietary sources of carbohydrates. More than one third 213 (34.9%) of girls think that fat free diet is energy free, more than half 343 (56.2%) of adolescent girls knew the dietary sources of fat. Almost three–fourths, 454 (74.4%), of participants think that eating too much fat can make them fat. About 304 (49.8%) of participants know the health consequence of lack of fiber from the diet (Table 6).

**TABLE 6:** Nutritional knowledge of adolescent girls attending secondary schools in Addis Ababa, Ethiopia, 2025.

Variables (*n* = 610)		Frequency (%)
Know least food group to consume	Fats, oils, and sweets	320 (52.5)
	Others^a^	290 (47.5)
Know most food group	Vegetables and fruits	200 (32.8)
	Others^b^	410 (67.2)
Is fat free energy free	Yes	213 (34.9)
	No	397 (65.1)
Know dietary sources of carbohydrates	Yes	328 (53.8)
	No	282 (46.2)
Know the dietary sources of fat	Yes	343 (56.2)
	No	267 (43.8)
Know the dietary sources of fibers	Yes	243 (39.8)
	No	367 (60.2)
Know the dietary sources of vitamin C	Yes	342 (56.1)
	No	268 (43.9)
Know the dietary sources of proteins	Yes	361 (59.2)
	No	249 (40.8)
Know that eating a lot of sugar, sweets, and sweet food can make you fat	Yes	460 (75.4)
	No	150 (24.6)
Know that eat too much fat you can become fat	Yes	454 (74.4)
	No	156 (25.6)
Know a low intake of fruit and vegetables will cause a health problem	Yes	190 (31.1)
	No	420 (68.9)
Know the health consequences of absence of fiber intake	Yes	304 (49.8)
	No	306 (50.2)
Know the health consequence of much consumption of salt	Yes	464 (76.1)
	No	146 (23.9)
Know the health consequences of much consumption of fatty foods	Yes	432 (70.8)
	No	178 (29.2)
Know eating fruit and vegetables every day is good for our bodies to prevent the occurrence of a certain disease	Yes	496 (81.3)
	No	114 (18.7)
Know that eating a lot of sugar, sweets, and sweet food is not good for your health	Yes	480 (78.7)
	No	130 (21.3)
Level of knowledge	Poor	307 (50.3)
	Good	303 (49.7)
^Mean knowledge score (mean ± SD)^	10.25 ± 3.04	

^a^Fruits and vegetables, bread, grains, rice, pasta, and cereals, and sweets and dairy and dairy products.

^b^Fats, oil and sweets bread, grains, rice, pasta, and cereals, and sweets and dairy and dairy products.

### 3.6 Dietary Diversity Score

**FIGURE 2:**
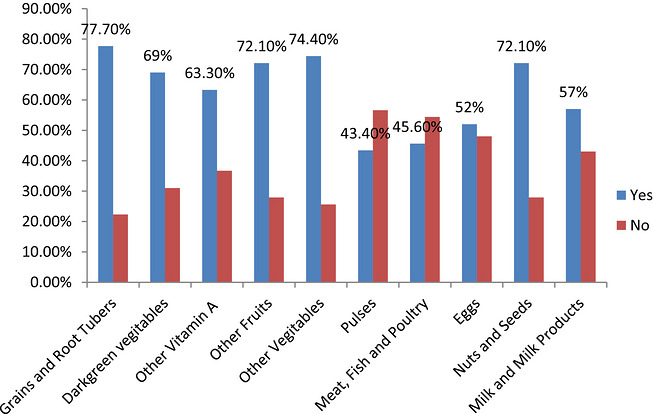
Types of food groups consumed by female adolescent girls in the last 24 h in Addis Ababa, Ethiopia, 2025.

The mean DDS was 6.48 ± 2.14 (mean ± SD). The majority, 474 (77.2%), of female adolescents have consumed cereals and grains in the last 24 h. Dark green vegetable consumption accounted for 421 (69%). Only 265 (43.4%) of girls consumed pulses. About 440 (72.1%) and 454 (74.4%) of female adolescents have consumed other fruits and vegetables respectively. Less than half 278 (45.6%) of participants have taken meat, fish, and poultry in the last 24 h. less than three fourth 440 (72.1%) of consumed nuts and seeds (Figure 2).

**FIGURE 3:**
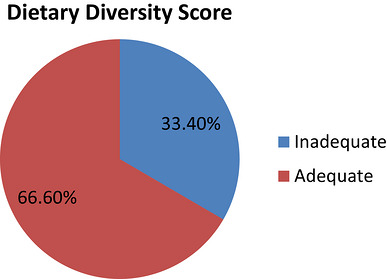
Pie chart on the prevalence of inadequate dietary diversity score among adolescent girls attending secondary schools in Addis Ababa, Ethiopia, 2025.

The prevalence of inadequate DD was found to be 204 (33.4%), with a 95%CI [29.66%, 37.14%] (Figure 3).

Meat, poultry, and fish (91.7%) were found to be the major food groups for attaining adequate dietary diversity (Table 7).

**TABLE 7:** The proportion of food group consumption tabulated with dietary diversity among high school.

Food groups	Inadequate DD *N* (%)	Adequate DD *N* (%)
Cereals, grains, and root tubers	138 (29.1)	336 (70.9)
Dark green vegetables	99 (23.5)	322 (76.5)
Vitamin A‐rich fruits and vegetables	71 (18.4)	315 (81.6)
Other fruits	96 (21.8)	344 (78.2)
Other vegetables	122 (26.9)	332 (73.1)
Pulses	22 (8.3)	243 (91.7)
Meat, poultry, and fish	23 (8.3)	255 (91.7)
Eggs	43 (13.6)	274 (86.4)
Nuts and seeds	117 (26.6)	323 (73.4)
Milk and milk products	41 (11.8)	307 (88.2)

*Note:* Adolescent girls in Addis Ababa, 2025 (*n* = 610).

**^Abbreviation:^ :**DD, dietary diversity.

### 3.7 Factors Associated With Inadequate DD

#### 3.7.1 Binary and Multivariable Logistic Regression

A total of 14 variables were found associated in the binary logistic regression. Some of the variables were maternal education, sweet food consumption, family history of chronic disease, home gardening, eating out of home, fathers’ education, nutritional education, meal frequency, and type of school. Those variables with *p* value less than 0.25 were exported to multivariable logistic regression.

Fourteen variables were exported from binary logistic regression to multivariable logistic regression. Four variables were associated in multivariable logistic regression. Adolescents with illiterate Mothers were 1.42 times more likely to have an inadequate DDS than those mothers with secondary and above education [adjusted odds ratio (AOR) = 1.42, 95%CI, (1.12–2.99)]. Meal frequency was found to be independent predictor of inadequate DD. Those girls who took more than three meals per day were 1.54 times more likely to have an inadequate DDS than those who took less than three meals [AOR = 1.54, 95%CI, (1.06–2.25)] The odds of having an inadequate DDS are 1.53 times higher for those adolescent girls who did not take nutritional education than those with nutritional education [AOR = 1.53, 95%CI, (1.35–1.80)]. Adolescent girls who did not consumes sweet foods/drinks were 1.71 times more likely to have an inadequate DDS than those with consumption [AOR = 1.71, 95%CI, (1.17–2.50)] (Table 8).

**TABLE 8:** Result of binary and multivariable logistic regression of factors associated with inadequate dietary diversity among high school adolescent girls in Addis Ababa, Ethiopia.

Variable		Dietary diversity		COR [95%CI]	*p* value	AOR [95%CI]	*p* value
		Inadequate (%)	Adequate (%)				
Age (years)	14–16	73 (36.7)	126 (63.3)	1.23 (0.86–1.76)	0.238	1.46 (0.98–2.15)	0.057
	17–19	131 (31.9)	280 (68.1)	1		1	
School type	Private	59 (28)	152 (72)	1	0.037	1	
	Government	145 (36.3)	254 (63.7)	1.47 (1.03–2.13)		1.31 (0.87–1.95)	0.184
Family size	<5	136 (35.2)	250 (64.8)	1.24 (0.87–1.77)	0.219	1.08 (0.74–1.59)	0.671
	≥5	68 (30.4)	156 (69.6)	1		1	
Maternal education	Cannot read and write	24 (39.3)	37 (60.7)	1.32 (1.11–2.35)	0.046	1.42 (1.12–2.99)	0.008^a^
	Can read and write	52 (40)	78 (60)	1.35 (0.87–2.10)	0.171	1.32 (0.73–2.36)	0.350
	Primary	44 (26.8)	120 (73.2)	0.74 (0.48–1.51)	0.186	0.69 (0.41–1.14)	0.150
	Secondary and above	84 (32.9)	171 (67.1)	1		1	
Fathers’ education	Cannot read and write	10 (28.6)	25 (71.4)	0.83 (0.38–1.79)	0.638	0.63 (0.24–1.61)	0.337
	Can read and write	50 (42.7)	67 (57.3)	1.55 (1.00–2.39)	0.048	1.13 (0.62–2.05)	0.681
	Primary	42 (29.2)	102 (70.8)	0.85 (0.55–1.31)	0.478	0.90 (0.54–1.48)	0.682
	Secondary and above	102 (32.9)	212 (67.5)	1		1	
Home gardening	Yes	65 (25.9)	186 (74.1)	1		1	
	No	139 (38.7)	220 (61.3)	1.82 (1.28–2.63)	0.001	1.32 (0.89–1.94)	0.162
Eating out	Yes	53 (27.3)	141 (72.7)	1		1	
	No	151 (36.3)	265 (63.7)	1.52 (1.04–2.22)	0.029	1.09 (0.72–1.65)	0.661
Breakfast before school	Yes	116 (30.0)	271 (70)	1		1	
	No	88 (39.5)	135 (60.5)	1.54 (1.09–2.17)	0.017	1.36 (0.92–2.00)	0.114
Nutritional education	Yes	45 (23.6)	146 (76.4)	1		1	
	No	159 (37.9)	260 (62.1)	2.00 (1.35–2.94)	0.001	1.53 (1.35–1.80)	0.003^a^
More sweet food/drink consumption	Yes	91 (27.9)	235 (72.1)	1		1	
	No	113 (39.8)	171 (60.2)	1.72 (1.22–2.42)	0.002	1.71 (1.17–2.50)	0.006^a^
Snacking	Yes	159 (37.2)	268 (62.8)	1.81 (1.23–2.68)		2.17 (0.93–3.31)	0.294
	No	45 (24.6)	138 (75.4)	1	0.003	1	
Long stay on social media	Yes	102 (30)	238 (70)	1	0.043	1	0.170
	No	102 (37.8)	168 (62.2)	1.43 (1.01–1.96)		1.29 (0.89–1.88)	
Family chronic disease	Yes	35 (26.1)	99 (73.9)	0.64 (0.41–0.98)	0.043	0.70 (0.44–1.11)	0.185
	No	169 (35.5)	307 (64.5)	1		1	
Meal frequency	≥3	124 (41.1)	178 (58.9)	1.98 (1.40–2.79)	0.000	1.54 (1.06–2.25)	0.024^a^
	<3	80 (26)	228 (74)	1		1	

^a^Statistically significant.

Abbreviations: AOR, adjusted odds ratio; COR, crude odds ratio.

## 4 Discussion

The study was conducted to assess the magnitude of inadequate DDS among adolescents’ girls attending secondary schools in Addis Ababa. The prevalence of inadequate DDS was found to be 33.4%, with a 95%CI [29.66%, 37.14%]. The study was lower than a previous study conducted in Bangladesh (55.4%) [39], Ghana (84.7%) [40], Eastern Uganda (45.3%) [20], and Nifas Silk Lafto subcity, Addis Ababa (77.2%) [29]. Although the level of inadequate DD in this study is lower, it still indicates a considerable public health concern among urban adolescents. The relatively better DD in Addis Ababa may reflect an improved access to diverse foods in urban environments, increased exposure to nutrition information, and continuous public health interventions targeting adolescent nutrition. On the contrary, improved food availability in urban settings like Addis Ababa does not necessarily ensure optimal diet quality, as dietary choices are also shaped by knowledge, preferences, and socioeconomic constraints [41]. The finding was higher than a previous study done in Brukina Faso (13.4%) [42]. The possible difference could be due to variation in contextual factors, including socioeconomic disparities, cultural dietary practices, and methodological variations (food group classification and cutoff points) [43]. This study showed that the mean DDS was consistent with other studies conducted in Tehran [44] and Iran [45]. This similarity may reflect the influence of urban food environments, where access to markets increases food options but does not always ensure balanced consumption [46]. The mean DDS was found to be higher than previous findings from Adama town [47] and Woldia, northern Ethiopia [9]. The observed variation could be due to the ongoing rural–urban disparities in food access and affordability [48].

The current study showed that the majority of participants consumed grains/roots and tubers, whereas pulses are the least consumed food groups in the current study. These dietary patterns may lead to micronutrient deficiencies even though there is adequate caloric intake [49]. The observed dietary pattern, characterized by high consumption of grains, roots, and tubers alongside relatively lower intake of diverse nutrient‐rich foods, shows a typical urban food environment of Addis Ababa. Staple foods can be widely available and relatively affordable, making them the main component of daily diets. On the contrary, consumption of fruits, vegetables, and other micronutrient‐rich food groups may be affected by food preference, economic constraints, seasonal variability, and rising food prices in urban settings. Additionally, rapid urbanization and the expansion of informal food markets may promote dependence on easily available, energy‐dense foods with limited DD. These contextual factors might entail the disproportionate consumption of different food groups and underline the need for targeted interventions to improve access to and utilization of diverse, nutrient‐rich foods among adolescent girls [50].

In this study, maternal education was an independent predictor of DD, underscoring its critical role in shaping adolescent nutrition. Educated mothers are more likely to have better awareness of balanced diets, influence household food allocation, and encourage healthy eating behaviors among their children [51]. Consistent findings were reported from a study done in South Africa [52]. This relationship may also reflect broader socioeconomic advantages, as education is often linked with better income and access to information [53].

Meal frequency was found to be an independent predictor of DDS. Consistent finding was reported from a study done in Indonesia [54]. However, increased frequency did not necessarily correspond to improved dietary quality. Research evidence from Bangladesh shows that adolescent girls often increase the frequency of snacking while skipping main meals, a pattern that may eventually lead to micronutrient deficiencies, including anemia [55]. The possible reason for the association might be household food insecurity, limited time due to school schedules, and cultural norms that may prioritize other family members during meal serving and distribution. The other possible reason could be due to a high nature of snaking sweet foods among adolescents, which are low in nutritional value [56].

Nutritional education/counseling is found to be independent predictor of inadequate DDS. Similarly, a consistent finding was reported from a study in Southern Ethiopia [11]. Adolescents with access to nutritional information had a better chance of diversifying their diet. Nutritional knowledge is a crucial determinant of food selection and dietary habits [57]. Research conducted in low‐income nations has shown that school‐based nutrition education initiatives substantially enhance DD and promote healthier eating behaviors [58].

Nonconsumption of more sweet drinks was found to be an independent predictor of inadequate DDS. The association between sweet food and drink consumption and DD observed in the current study differs from a previous finding in Jimma [59]. Adolescent girls who eat fewer sweet foods or drinks may come from lower socioeconomic households with limited access to diversified foods, which could result in a monotonous diet. On the contrary, increased access to processed meals and better purchasing power may be the reason for higher intake of sweetened foods in urban areas. Sweet food consumption and overall DDS may also be influenced by cultural factors and parental control over food choices. Similar results have been documented in other research, suggesting that a number of factors, such as social norms, the food environment, and income, have an impact on eating habits [60].

## 5 Limitation of the Study

The cross‐sectional nature of the study limits us to ascertain the exposure in the study. Use of 24 h dietary recall may not show the dietary pattern and is more prone to recall and social desirability bias.

## 6 Public Health Implications, Conclusion, and Recommendation

### 6.1 Public Health Implications

The current study underlines the urgent need to improve the DD among adolescent girls through targeted nutrition education and awareness programs. Strengthening maternal education and promoting regular and diversified meal patterns can positively impact dietary practices. Limiting consumption of sweet foods and sugar‐sweetened drinks should be emphasized in school and community interventions. These strategies can help reduce the risk of micronutrient deficiencies and support healthy growth and development in this population.

## 7 Conclusion

According to the current study, a considerable proportion of adolescent girls have inadequate DD, with diets largely dominated by staple foods and limited intake of diverse nutrient‐rich foods. Maternal education, meal frequency, nutrition education, and dietary habits related to sweet food and drink consumption were identified as key determinants. These findings underscore the need for targeted nutrition intervention programs focusing on improving dietary practices, strengthening school‐based nutrition education by including nutrition in school curriculum, and promoting household awareness to enhance adolescent nutrition. Addressing these factors is key to tackling intergenerational cycle of malnutrition, improve DD, and reducing the risk of micronutrient deficiencies among adolescent girls.

## 8 Recommendations

On the basis of the findings, the following recommendations were made as follows:

- The Addis Ababa Health Bureau should scale up nutritional education services by using different digital media platforms for adolescent girls.
- The Addis Ababa Health Bureau should promote reduced intake of sweets and sugar‐sweetened beverages through targeted behavior change interventions.
- The city government of Addis Ababa should work to improve availability, affordability, and access to diverse nutrient‐dense foods (urban agriculture, Sunday markets).
- The city government of Addis Ababa should expand the SFPs to secondary schools to improve dietary quality and adequacy.
- The Education Office of Addis Ababa City government should empower and educate women.
- The education office of Addis Ababa city government should work on integrating nutrition education into the school curriculum to ensure sustained knowledge and behavior change.
- The school directors should collaborate with teachers to expand school health clubs for improving nutritional knowledge and dietary practice.
- The health professionals in Addis Ababa should educate adolescents about balanced diet for healthy dietary choices.
- Researchers should conduct a longitudinal study to explore more factors.

## Author Contributions

**Bekalu Getachew**: conceptualization, methodology, project writing – original draft. **Aragaw Gizaw**: data curation, software and editing of the manuscript. **Habtamu Molla**: data curation, project administration and write up. **Ruth Tilahun**: tool support, investigation, writing – review and editing. **Abdu Oumer**: data curation, investigation, methodology and manuscript review with editing.

## Funding

The authors have nothing to report.

## Ethics Statement

The research was carried out in accordance with the Declaration of Helsinki and was approved by Addis Ababa city government Public Health research Ethical review board.

## Conflicts of Interest

The authors declare no conflicts of interest.

## Data Availability

Data will be available upon request of the corresponding author.
